# Insights into antibiotic resistomes from gut metagenome-assembled genomes of the free-range pigs

**DOI:** 10.1128/spectrum.02407-25

**Published:** 2026-02-27

**Authors:** Xihui Dai, Huan Liu, Xue Bai, Diyan Li, Tao Wang, Hang Zhong, Huailiang Xu, Jing Sun

**Affiliations:** 1 A College of Life Science, Sichuan Agricultural University, Ya 'an, China; 2 Department of Emergency, Ruijin Hospital, Shanghai Jiao Tong University School of Medicine 56694, Shanghai, China; 3 School of Pharmacy, Chengdu University 74707, Chengdu, China; 4 Institute of Bioengineering, ChongQing Academy of Animal Sciences 580592, Chongqing, China; 5 National Center of Technology Innovation for Pigs, Chongqing, China; 6 ChongQing Academy of Animal Sciences 580592, Chongqing, China; Nanjing Agricultural University, Nanjing, China

**Keywords:** pig gut microbiome, antibiotic resistance, metagenome-assembled genomes, regional variation, mobile genetic elements

## Abstract

The pig gut microbiome serves as a reservoir for antibiotic resistance genes (ARGs), which pose a threat to public health and environmental safety. To investigate the presence of ARGs carried by free-range pigs, which have frequent contact with humans and their environment, we characterized the resistome of the pig gut microbiome through metagenomic sequencing of fecal samples from 120 pigs across four provinces in China (Yunnan, Guizhou, Sichuan, and Jiangsu). By constructing metagenome-assembled genomes (MAGs) and gene catalogs, we explored the microbial community structure and ARG distribution. Our analysis revealed a highly diverse array of ARGs, particularly those conferring resistance to multidrug, glycopeptide, peptide, and tetracycline antibiotics. Bacillota A and Actinomycetota were the dominant phyla across samples. However, notable regional differences in microbiota composition and resistance profiles were observed. These differences were likely influenced by local farming practices and environmental conditions. Guizhou harbored 11 unique ARG types, followed by Sichuan (seven), which showed region-specific resistome signatures. *Escherichia coli* and other microbial taxa were closely linked with ARG abundance, suggesting potential vectors for horizontal gene transfer. Analysis of mobile genetic elements (MGEs) further supported this, revealing a strong linear correlation between MGE and ARG abundance, with transposase elements particularly associated with multidrug ARGs. These findings highlight the central role of MGEs in ARG dissemination and underscore the need for targeted strategies to curb antibiotic resistance in livestock systems. Regional variation in resistome profiles further emphasizes the influence of local agricultural practices on resistance dynamics.

**IMPORTANCE:**

The growing prevalence of antibiotic resistance poses a significant global health threat, making it imperative to trace the origins and transmission routes of ARGs. This study delivers a comprehensive genomic reference for the porcine gut microbiota and clarifies how regional farming practices shape distinct resistome profiles. Integrating these data with analyses of mobile genetic elements and microbial hosts reveals the complex interplay among host, microbiota, and environment, thereby extending current knowledge of the pig gut ecosystem. These findings provide an evidence-based foundation for targeted surveillance and intervention strategies to curb antibiotic resistance in livestock and safeguard public health.

## INTRODUCTION

Antibiotic resistance has emerged as one of the most urgent global health crises of the 21st century (1). In 2019 alone, nearly five million deaths worldwide were associated with drug-resistant infections (2), and the World Health Organization (WHO) has warned that this number could rise to 10 million annually by 2050 without global intervention (3, 4). Among the many contributors to this growing threat, livestock, particularly pigs, are increasingly recognized as significant reservoirs of antibiotic resistance genes (ARGs) (5). These genes can enter the food chain and the environment, where they may ultimately impact human health through direct or indirect transmission (6).

Domestic pigs, one of the world’s most widely farmed animals (7), play a vital role not only in food production but also as a model organism for biomedical research (8–10). The pig gastrointestinal tract hosts trillions of bacteria, forming a complex and dense microbial ecosystem that is essential for host immunity, metabolism, and development (11). This microbiota also comprises a vast reservoir of ARGs, collectively referred to as the resistome (12), which can be mobilized under selective pressure. ARGs originating from the pig gut may be transferred to humans via contaminated meat products, animal waste, or environmental pathways (13). Free-range pigs, which are raised in more natural and open environments (14). This closer proximity to human settlements and environmental elements increases the likelihood of ARGs being transmitted from pigs to humans through direct contact, contaminated water sources, or soil (15). Additionally, free-range pigs are often subject to different agricultural practices, including smaller-scale farming and potentially less stringent antibiotic use regulations, which can influence the prevalence and diversity of ARGs in their gut microbiota (16, 17). These factors collectively contribute to the unique significance of studying free-range pigs to understand the transmission dynamics and public health risks associated with ARGs.

However, little is known about how ARGs are structured and transmitted in free-range pigs, which frequently interact with both humans and the environment. Metagenomics, which allows for direct sequencing of DNA from environmental samples, has transformed our ability to investigate microbial communities and their functional capabilities (18). In pigs, metagenomic approaches have been used to link gut microbiota to traits such as feed efficiency (19, 20), growth performance (21), and increased resistance to diarrhea in early-weaned piglets (22). In particular, the reconstruction of metagenome-assembled genomes (MAGs) has expanded our understanding of microbial diversity and function, offering a high-resolution view of bacterial composition, host associations, and gene flow within ecosystems (23–25). Recent applications of MAG-based approaches in pig gut microbiomes have revealed novel ARGs, virus-based interactions, and taxon-specific functions (5).

Despite this progress, the impact of geography and farming practices on ARG prevalence and transmission in pig gut microbiota remains underexplored. Microbial communities and resistomes are known to vary with husbandry methods, diet, and environmental conditions (5, 26), suggesting that local farming practices could significantly influence resistance profiles. Understanding these patterns is needed to tailor interventions that address regional risk factors.

In this study, we analyzed the pig gut resistome by generating MAGs and gene catalogs from 120 fecal samples collected across four distinct Chinese provinces. Our aim was to characterize the structure and distribution of ARGs, identify microbial hosts and mobile genetic elements involved in their spread, and evaluate regional variations in resistance profiles. By integrating taxonomic, functional, and ecological insights, we provide a foundation for managing antibiotic resistance in swine farming and mitigating its broader public health impact.

## MATERIALS AND METHODS

### Sample collection and DNA extraction

We collected 120 pig fecal samples across four provinces in China—Yunnan, Guizhou, Sichuan, and Jiangsu—in 2021, with 30 samples collected from each province. Detailed metadata, including basic pig characteristics, sample sources, specific sampling times, geographic locations, elevation, and Global Positioning System (GPS) coordinates, are provided in Table S1. All pigs were healthy, with no signs of disease or infection, and had not received any antibiotic treatment or medical interventions involving antibiotics. Ten samples were collected from each village, with a minimum distance of 10 km between adjacent villages.

All samples were collected uniformly between 8:00 and 10:00 AM. Immediately after defecation, a sterile swab was used to remove surface contaminants from fresh feces, such as soil, hair, and food residues. Subsequently, approximately 1–5 g of fecal samples were collected from the middle layer (an area not exposed to the external environment) using a sterile spoon and placed into a sterile collection tube. All samples were immediately stored at −80°C to preserve microbial DNA integrity. Genomic DNA was extracted using the QIAamp Fast DNA Stool Mini Kit (Qiagen, Hilden, Germany), following the manufacturer’s protocol. DNA quality and concentration were assessed using a NanoDrop spectrophotometer and agarose gel electrophoresis.

### Metagenomic sequencing

Genomic DNA was fragmented to an average length of approximately 350 bp for paired-end library construction. Adapters containing full sequencing primer sites were ligated to blunt-ended fragments. Paired-end sequencing was performed on the Illumina NovaSeq 6000 by Beijing Novogene Technology Co., Ltd. All sequencing data have been deposited in the NCBI database under accession number PRJNA1039141.

### Metagenomic assembly and quality control

Raw sequencing data were processed using the Majorbio Cloud Platform (https://www.majorbio.com). Paired-end Illumina reads underwent adapter trimming and removal of low-quality reads (< 50 bp or quality score < 20) using fastp (version 0.23.0) (27). Additional quality filtering was performed using SeqPrep (https://github.com/jstjohn/SeqPrep) and Sickle (version 1.33, https://github.com/najoshi/sickle). Host-derived sequences were removed by aligning clean reads to the pig genome (Sscrofa11.1) using BWA (28) (version 0.7.9a, http://bio-bwa.sourceforge.net). High-quality reads were then assembled using MEGAHIT (29) (version 1.1.2).

### Metagenomic binning and quality control of MAGs

Metagenomic binning was performed on each sample using Metabat2 (version 2.12.1) (30), MaxBin2 (version 2.2.5) (31), and CONCOCT (version 0.5.0) (32). Non-redundant genome bins were consolidated using DAS Tools (version 1.1.0) (33). To improve genome completeness and reduce contamination, RefineM (version 0.0.24) (34) was used to remove contigs with inconsistent genomic features such as GC content, tetranucleotide signatures, and taxonomic assignment. Genome completeness and contamination were evaluated with CheckM (version 1.0.12) (35), using lineage-specific marker genes. Medium-quality MAGs were defined as those with ≥50% completeness and <10% contamination, and high-quality MAGs as those with ≥90% completeness and ≤5% contamination.

MAGs with ≥50% completeness and <10% contamination were retained (34). Pairwise comparisons of average nucleotide identity (ANI) were conducted using Mash (36). Redundant genomes (ANI ≥99%) were dereplicated using dRep (version 3.4.2) (37), keeping only the highest-quality MAG from each cluster. Genome coverage was estimated using CoverM (version 0.6.1), and taxonomic classification was assigned based on 120 single-copy marker proteins using GTDB-Tk (version 2.3.0) (38).

### Gene prediction and functional annotation

Open reading frames were predicted using Prodigal (version 2.6.3) with the -p meta option to accommodate the metagenomic context (39). Predicted genes were annotated using several databases: KEGG (built from August 2023), eggNOG (built from August 2023), and CAZy (version 8, August 2023).

### ARGs and MGE annotation

ARGs were characterized through the alignment of non-redundant genes against the CARD (Comprehensive Antibiotic Resistance Database) using DIAMOND‌ (40) (version 3.0.9, e-values ≤ 1E−5). MGE annotation was performed using Diamond (40) [version MGEs90 (https://github.com/KatariinaParnanen/MobileGeneticElementDatabase), e-values ≤ 1E−5].

### Statistical data analysis

Data visualization and statistical analysis were conducted using multiple tools. Maps, violin plots, clustering heatmaps, pie charts, bar charts, and correlation scatter plots were generated with the Microbioinformatics platform (https://www.bioinformatics.com.cn) and ImageGP 2 (41) (https://www.bic.ac.cn/ImageGP/); Sankey diagrams and correlation heatmaps for MAG species were created with Python (v2.7.10); and UpSet Venn diagrams, dual-matrix correlation heatmaps, KEGG histograms, COG annotation classification statistics, antibiotic resistance gene prediction classification statistics, and gene potential migration analysis diagrams were produced with the Majorbio platform(https://v.majorbio.com/projectcenter/overview).

To explore the relationship between microbial community composition and environmental variables, Redundancy Analysis (RDA) was performed using the Wekemo Bioincloud platform (42), and permutation multivariate analysis of variance (PERMANOVA) was conducted using the phych and ggplot2 packages in R. The correlation between MGE types and ARG categories was analyzed using Spearman’s correlation coefficient, with significance levels indicated by *P*-values (* *P* < 0.05, ** *P* < 0.01, *** *P* < 0.001). The R scripts for reproducible analysis are available at: https://github.com/13887740018/Pig-MAG/tree/master.

## RESULTS

### Assembly of 4,165 MAGs from pig gut microbiome sequencing data

**Fig 1:**
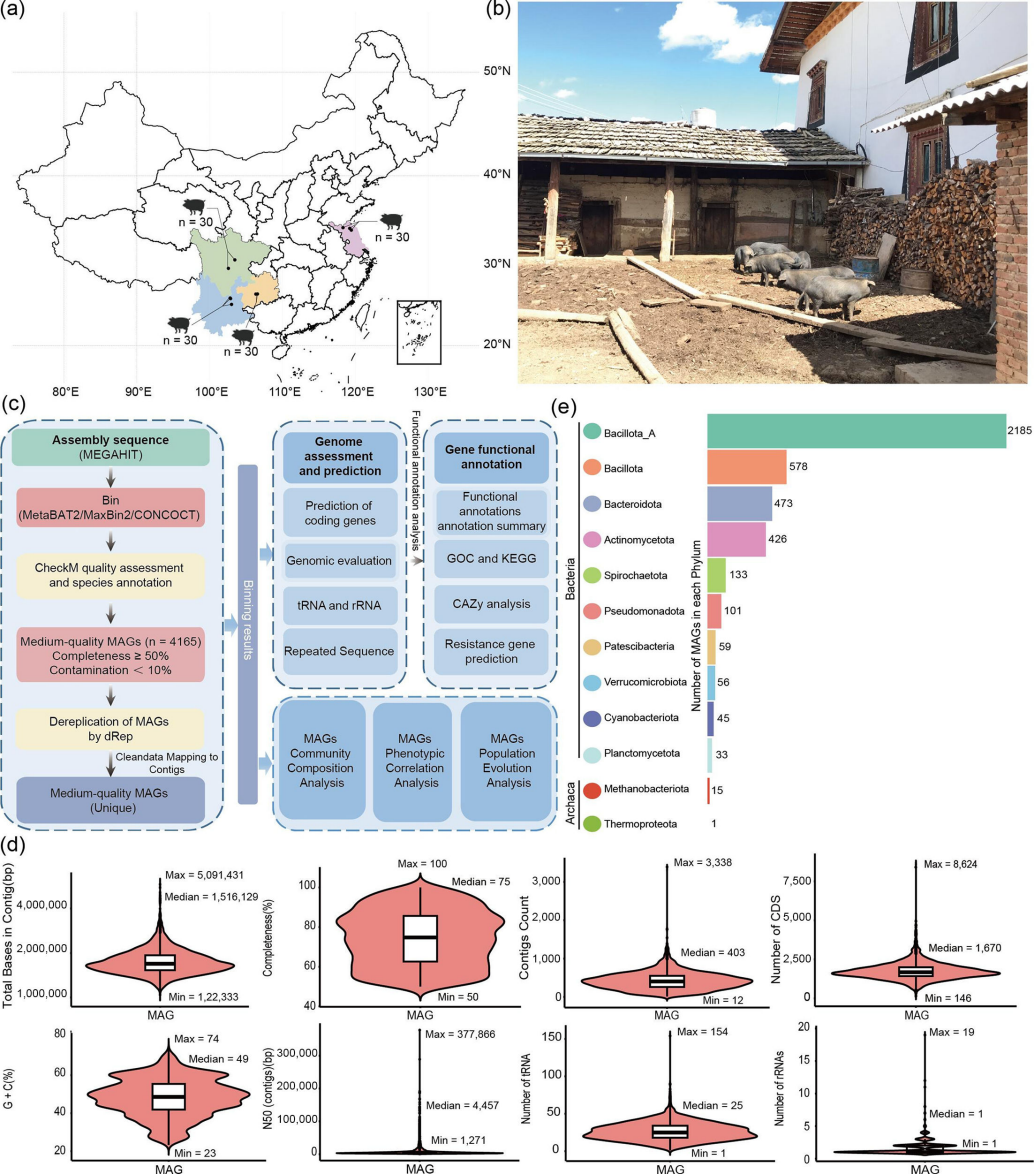
Workflow and summary statistics of metagenomic assembly and MAG classification. (**a**) Geographic locations of sample collection sites across four Chinese provinces. (**b**) An actual sampling environment from villages in Yunnan. Fecal samples were taken from the pigs' farms that belong to the villagers. These farms are generally placed close to the villagers' houses. (**c**) Overview of genome assembly, gene catalog construction, and functional annotation pipeline. (**d**) Assembly statistics for high- and medium-quality MAGs, including total genome length, completeness, number of contigs, CDS count, GC content, N50, and numbers of tRNA and rRNA genes. (**e**) Taxonomic composition of MAGs at the phylum level. Each colored circle represents the number of MAGs assigned to each phylum, listed in descending order.

To gain a comprehensive understanding of ARGs in the pig gut microbiome and elucidate the transmission of resistance genes from animals to humans, a total of 120 fecal samples were collected from pigs in four Chinese provinces—Yunnan, Sichuan, Guizhou, and Jiangsu (*n* = 30 per province)—for metagenomic sequencing and subsequent assembly of MAGs (Fig. 1a and c; Table S1). The samples were primarily obtained from free-range pigs, and the farming locations were in close proximity to human settlements (Fig. 1b; Fig. S1). This sampling strategy enabled us to better understand the transmission of ARGs from animals to humans. Shotgun metagenomic sequencing of these samples yielded a combined total of 1.53 Tb of raw data, averaging 12.78 Gb per sample (Table S1). After removing host-derived sequences, the average per-sample data were reduced to 11.63 Gb (Table S1).

Based on quality criteria from the Genomic Standards Consortium, a total of 4,165 MAGs were reconstructed, meeting medium- to high-quality thresholds (completeness ≥ 50%, contamination < 10%), with an average of 35 MAGs per sample (Table S2). The mean completeness was 74.5%, and the mean contamination was 2.8%. Of these, 636 MAGs met the more stringent high-quality standard (completeness ≥ 90%, contamination ≤ 5%) (Table S2). These MAGs were evaluated using multiple genome characteristics, including total contig length, contig count, number of contigs, GC content, N50 length, and number of rRNA and tRNA genes (Fig. 1d; Table S2).

Taxonomic classification of the 4,165 MAGs was performed using the Genome Taxonomy Database (GTDB). The analysis assigned 632 MAGs at the species level, 2,097 at the genus level, 3,188 at the family level, 3,876 at the order level, and all 4,165 at the class level (Table S3). Overall, the MAGs represented 20 bacterial phyla (*n* = 4,165) and two archaeal phyla (*n* = 16) (Table S3). The top five bacterial phyla (GTDB taxonomy) were Bacillota_A (2,185 MAGs; 52.46%), Bacillota (578 MAGs; 13.88%), Bacteroidota (473 MAGs; 11.36%), Actinomycetota (426 MAGs; 10.23%), and Spirochaetota (133 MAGs; 3.19%) (Fig. 1e). Among the archaeal MAGs, 15 were assigned to Methanobacteriota and 1 to Thermoproteota.

### Taxonomic annotation of MAGs

**Fig 2:**
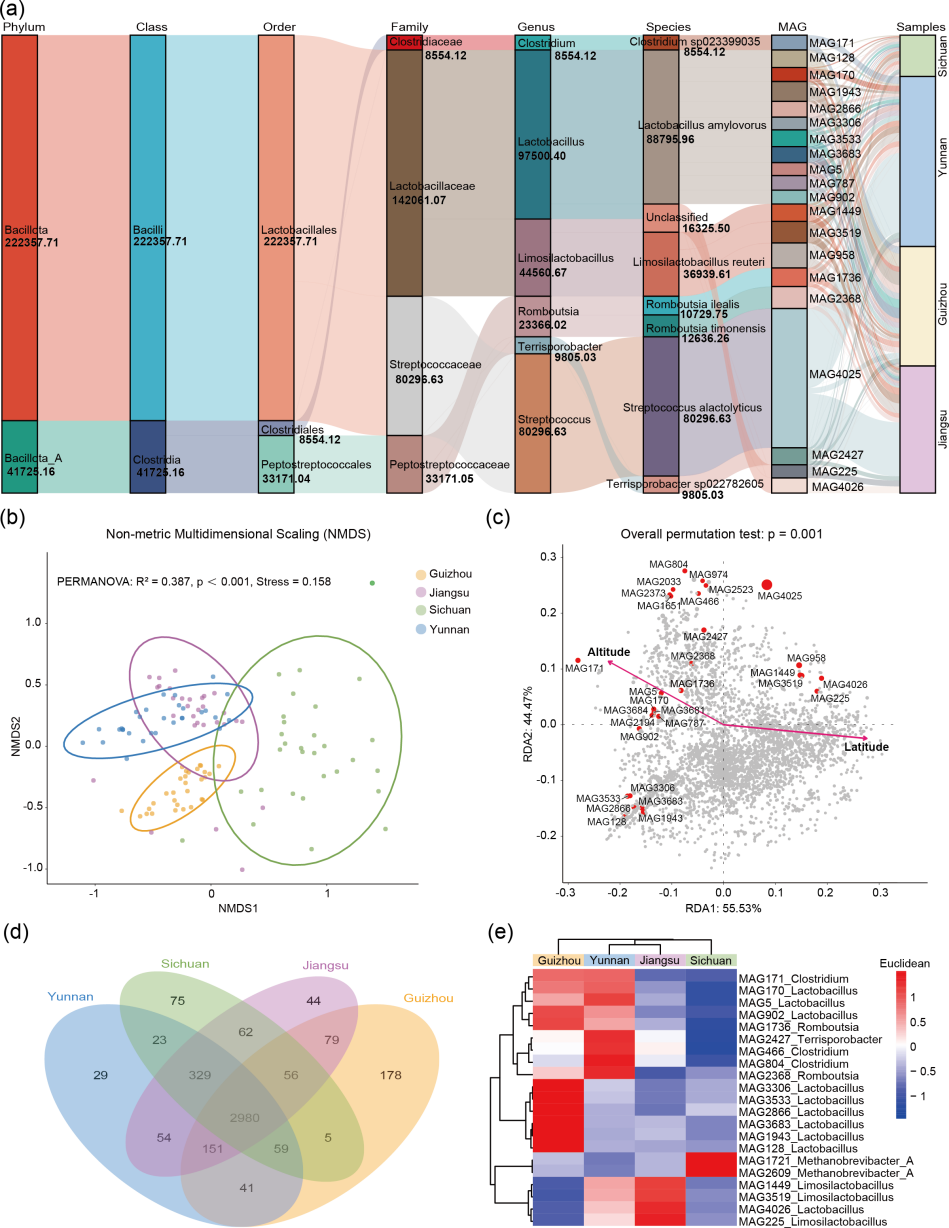
Community composition and geographic distribution of pig gut MAGs. (**a**) Sankey diagram displaying the abundance and taxonomic relationships of the top 20 MAGs. Columns represent taxonomic levels, with colored bands indicating species and band widths proportional to abundance. Gray connecting lines between bands illustrate the correspondence among species and samples across different levels. The numbers below each species represent their respective abundance values. (**b**) Non-metric multidimensional scaling (NMDS) analysis of pig gut microbiota communities across four Chinese provinces. The ordination plot displays the sample distribution based on Bray-Curtis diversity of microbial community composition. Each point represents an individual sample, with color indicating its provincial origin. Ellipses denote the 95% confidence intervals for each cluster. (**c**) Relationship diagram between MAGs and environmental factors based on RDA (Redundancy Analysis). The RDA1 axis explains 55.53% of the variance, while the RDA2 axis accounts for 14.47% of the variance. Arrows indicate the direction of influence from environmental factors (elevation and latitude). Red dots represent distinct MAGs, whose distribution reflects correlations with environmental factors. (**d**) Venn diagram illustrating the number of MAGs shared or unique across the four provinces. (**e**) Heatmap showing the relative abundance and clustering of the top 10 shared MAGs from each province. MAGs are annotated to the genus level.

To characterize the structure and regional distribution of the pig gut microbiota, we employed the TPM (Transcripts Per Million) normalization method and visualized the taxonomic abundance of microbes using Sankey diagrams and heatmaps across samples and hierarchical taxonomic levels (Table S3). At the phylum level, Bacillota ranked first in average relative abundance among the top 20 MAGs and constituted a prevalent component of the microbial community (Fig. 2a; Table S3). As the taxonomic resolution increased to genus and species, the Sankey diagram displayed more complex branching. Some species showed regional enrichment; for instance, MAG1943 (*Lactobacillus amylovorus*) was enriched in Guizhou Province.

PERMANOVA confirmed that geographic origin (province) significantly shaped microbial community structure (R² = 0.387, *P* < 0.001), accounting for 38.7% of the total variance (Fig. 2b). RDA further linked community variation to environmental gradients, revealing a significant model (*P* = 0.001) in which altitude emerged as the primary factor influencing MAG distribution (Fig. 2c). Specifically, MAG804 (*Clostridium*) and MAG4,025 (*Terrisporobacter*) were enriched in high-altitude environments, whereas MAG171 (*Romboutsia*) and MAG5 (*Lactobacillus*) were associated with low-altitude conditions (*P* = 0.001). Latitude also contributed moderately to community assembly. These findings collectively underscore the important role of environmental factors in structuring the pig gut microbiota. To examine the overlap and regional specificity of MAGs across the four provinces, a Venn diagram was constructed (Fig. 2d). A total of 2,980 MAGs were shared across all four provinces, forming a core microbiome consistently present across regions. In contrast, each province also had unique MAGs: Yunnan had 29; Sichuan, 75; Guizhou, 178; and Jiangsu, 44.

To explore the composition and abundance of shared MAGs in more detail, we selected MAGs based on their relative abundance ranking (top 10 per province) and generated a clustering heatmap (Fig. 2e). The heatmap revealed distinct microbial patterns across provinces. These top MAGs were annotated to specific genera, enabling precise interregional comparisons. The majority were affiliated with Lactobacillus (10 MAGs; 45.45%), followed by *Limosilactobacillus* (4 MAGs; 18.18%), *Clostridium* (3 MAGs; 13.64%), and *Methanobrevibacter_A* (2 MAGs; 9.09%). At the provincial level, Sichuan exhibited a high abundance of *Methanobrevibacter_A*, Jiangsu had elevated levels of *Limosilactobacillus*, Yunnan showed a high prevalence of *Clostridium*, and Guizhou was dominated by *Lactobacillus*. These results reflect both shared and region-specific features of the pig gut microbiome.

### Functional characteristics of the pig gut microbial gene catalog

To explore the functional capacity of the pig gut microbiome, gene annotations were performed using three major databases: the Cluster of Orthologous Groups (COG), the Kyoto Encyclopedia of Genes and Genomes (KEGG), and the Carbohydrate-Active enZYmes (CAZy) database. From the 4,165 medium- and high-quality MAGs, a total of 7,240,605 predicted protein-coding genes were obtained (Table S4).

**Fig 3:**
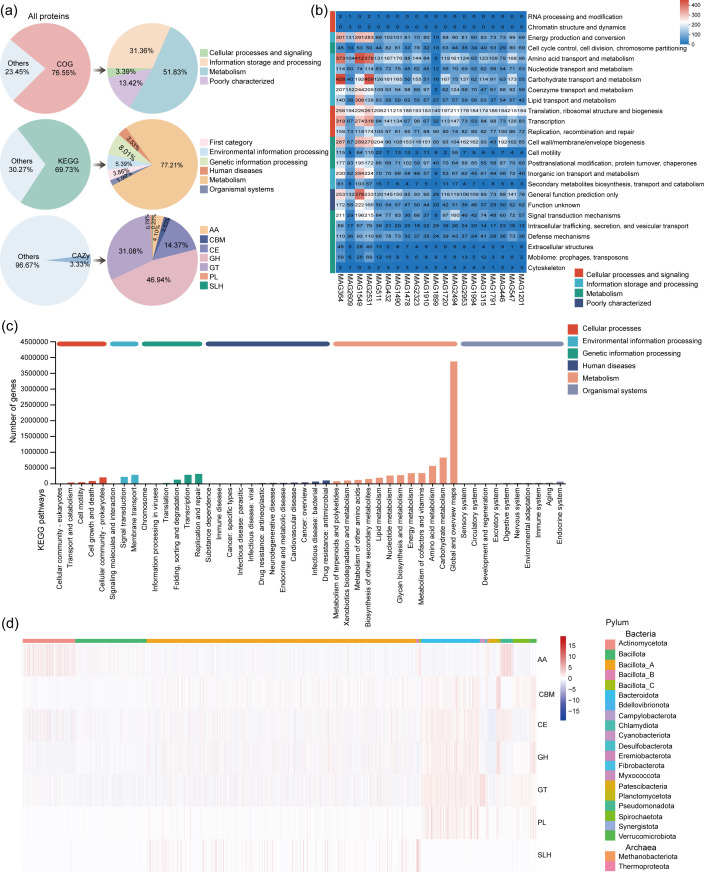
Functional annotation of pig gut microbial genes based on COG, KEGG, and CAZy databases. (**a**) Summary of gene annotations: percentage of predicted proteins assigned to COG, KEGG, or CAZy databases. (**b**) Heatmap of COG functional classifications for 20 high-quality MAGs. The left row represents COG categories corresponding to specific functions, while the columns represent MAGs. The numbers indicate the number of genes per function. (**c**) KEGG pathway classification bar chart. The y-axis shows level 2 pathways; bar lengths indicate the number of annotated genes. Bar colors reflect level 1 classifications. The rightmost bar shows gene counts aggregated at level 1. (**d**) Heatmap of CAZy gene distributions across bacterial phyla. Columns represent taxa, and rows represent CAZy categories.

Among these predicted proteins, 76.55% (5,542,892) were assigned at least one COG function, 69.73% (5,048,950) were assigned at least one KEGG function, and 3.33% (240,782) were assigned at least one CAZy function (Fig. 3a; Table S4). The COG annotations categorized the genes into four overarching functional classes: cellular processes and signaling, information storage and processing, metabolism, and poorly characterized functions. These were further divided into 25 specific COG categories, with cellular processes and signaling being the most prevalent (Table S4).

To examine functional variation across microbial taxa, we analyzed the COG annotations of 20 high-quality MAGs (completeness ≥ 95%, contamination ≤ 0.1%). These MAGs displayed distinct functional profiles (Fig. 3b). For example, MAG364 and MAG2531 (both Pseudomonadota) possessed a high proportion of genes involved in amino acid transport and metabolism as well as carbohydrate transport and metabolism.

KEGG annotations provided further insight into metabolic functions (Fig. 3c). Protein-coding genes were grouped into six major metabolic categories, with metabolism representing the major class (31.06% of total proteins). Within this group, genes associated with global and overview maps and carbohydrate metabolism were particularly enriched (Table S4). These findings align with the COG results, reinforcing the central role of metabolism, especially carbohydrate metabolism, as a key functional attribute of the pig gut microbiome. The prevalence of genes in global metabolic pathways also highlights the integrated and cooperative nature of microbial functions within the gut environment.

Annotation using the CAZy database revealed a diverse and taxonomically variable array of carbohydrate-active enzymes. Among the 240,782 proteins identified, glycoside hydrolases (GH) were the major (113,020), followed by glycosyl transferases (GT; 74,835), carbohydrate-binding modules (CBM; 6,099), carbohydrate esterases (CE; 34,608), polysaccharide lyases (PL; 1,876), auxiliary activity proteins (AA; 9,870), and cellulosome modules (SLH; 474) (Table S4). Notably, these enzyme classes were unevenly distributed across taxa, with Bacillota_A and Bacteroidota exhibiting a high enrichment in GH and GT genes (Fig. 3d), suggesting that these groups may play a key role in carbohydrate degradation within the pig gut ecosystem (43).

### Antibiotic resistance gene profiling

To investigate the landscape of ARGs in the pig gut, we profiled ARGs across 636 high-quality MAGs (≥ 90% complete, ≤ 5% contaminated) from 120 fecal samples. ARG identification was performed using the Comprehensive Antibiotic Resistance Database (CARD). In total, 152 distinct ARG types spanning 35 antibiotic resistance classes were detected across the MAGs (Table S5). These findings align with previous research linking antibiotic use in pigs to the numbers and diversity of ARGs within their gut microbiome (44).

**Fig 4:**
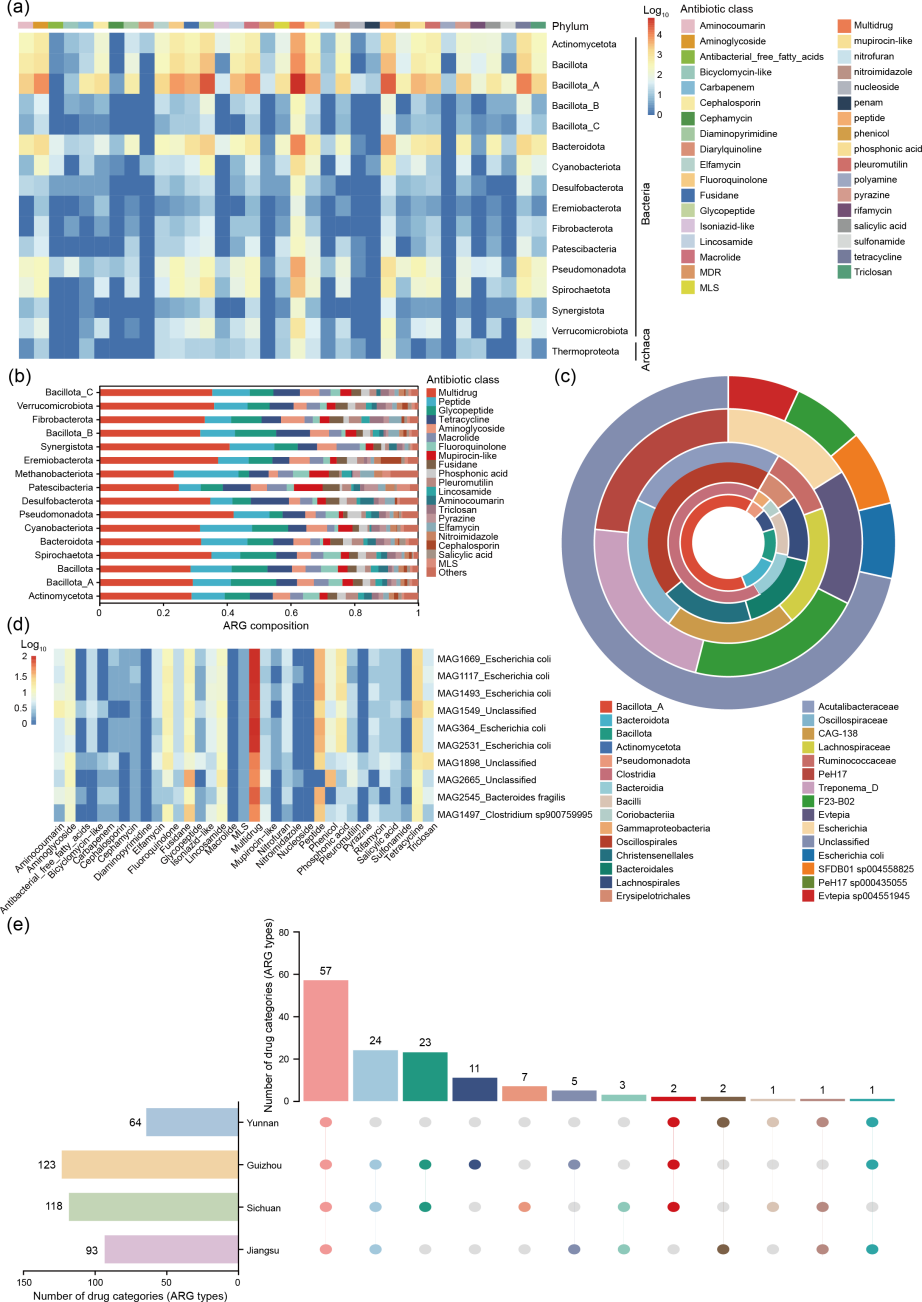
Antibiotic resistome profiling of pig gut MAGs. (**a**) Heatmap of ARG number across MAGs. Color intensity (blue to red) indicates ARG count; the top color bar denotes antibiotic class. (**b**) Stacked bar chart showing distribution of ARG classes across bacterial phyla. The y-axis lists phyla, and the x-axis shows the proportion of annotated ARGs. (**c**) Circular plot of ARG distribution across taxonomic levels. (At each taxonomic rank, only the top five taxonomic units exhibiting the greatest number of resistance genes are presented.) Innermost to outermost rings represent phylum to species level; colors indicate taxonomic groups. (**d**) Heatmap of the top 10 MAGs with the highest ARG counts, categorized by antibiotic class. Rows represent MAGs; columns represent ARG classes. (**e**) Upset plot showing the overlap and distribution of ARG types (drug classes) across four provinces. Vertical bars represent shared ARG types, with a dot matrix below indicating group membership. Horizontal bars show total ARG type counts per province.

Among the bacterial phyla, Bacillota and Actinomycetota harbored the greatest number of ARGs. Notably, ARGs were also identified in 16 archaeal taxa, including *Thermoproteota* (Fig. 4a). The most common resistance genes were those conferring resistance to multidrug agents, peptides, glycopeptides, and tetracyclines (Fig. 4b). The genera *PeH17*, *Treponema_D*, and *F23-B02* contained the highest number of ARGs per genome, among taxa represented by at least five MAGs (≥ 5 genomes per genus) (Fig. 4c). The predominance of multidrug resistance genes underscores the potential for broad-spectrum resistance among pig gut microbes, posing challenges for effective antibiotic treatment in livestock (45). Importantly, our analysis revealed a high prevalence of ARGs within the pig intestinal microbiome, with many MAGs containing multiple ARGs (Table S5). One particularly concerning example was *Escherichia coli* MAG1,669, which possessed 84 unique ARGs covering 27 different resistance classes (Fig. 4d; Table S5). This strain exhibited resistance to a broad array of antibiotics, including multidrug agents, peptides, glycopeptides, tetracyclines, phosphonates, aminoglycosides, and fluoroquinolones. The diversity and number of ARGs in this *E. coli* strain suggest high adaptability and an elevated risk for horizontal gene transfer and dissemination of resistance traits (46).

Several ARG-rich MAGs also belonged to microbial taxa with recognized probiotic potential. For example, notable ARGs were identified in *Bifidobacterium pseudolongum* (MAG2,687), *Anaerobutyricum hallii* (MAG464), and others (Table S5). While ARGs may facilitate the survival and future cultivation of these strains, their presence also raises concerns regarding safety and the potential transfer of resistance genes to pathogenic microbes within the host gut (47).

To further explore geographic variation in antibiotic resistance, we performed pairwise analyses of ARGs in province-specific high-quality MAGs from pig gut microbiomes of Guizhou, Yunnan, Sichuan, and Jiangsu provinces. Guizhou was observed to have a high total number of ARGs and a broad diversity of resistance categories among the provinces studied (Table S6). Region-specific ARGs were also identified: Guizhou had eleven unique ARG types, Sichuan had seven, while Jiangsu and Yunnan had no unique types of antibiotic-resistant bacterial communities (Fig. 4e; Table S6).

### Mobile genetic elements

The association between ARGs and MGEs is a critical factor influencing the potential for horizontal gene transfer within microbial communities (48). To assess the transferability of ARGs in the pig gut microbiome, we analyzed MGE content across the 636 high-quality MAGs.

On average, each MAG contained 32.18 MGE-related genes (Table S4). A total of 20,466 MGE-related genes were identified by aligning protein sequences from the gene catalog against the MGE database (Table S7). These genes were grouped into 45 distinct MGEs and categorized into five functional types: transposases, integrases, recombinases, conjugative transfer proteins, and transposons (Table S7). Among these, transposase genes were the predominant type, comprising 91.84% (*n* = 18,795) of all identified MGEs. Their prevalence underscores their significant role in facilitating the horizontal spread of ARGs across microbial populations.

To explore regional differences in ARG transmission mechanisms, we examined MGE profiles in province-specific high-quality MAGs from each of the four provinces (Yunnan, Guizhou, Sichuan, and Jiangsu) (Table S8). Transposase and XerD (a site-specific tyrosine recombinase) were the dominant MGE subtypes across all provinces. Our analysis revealed that Sichuan, Guizhou, Yunnan, and Jiangsu contained 21, 17, 14, and 14 MGE subtypes, respectively. Among these, Guizhou had five unique subtypes, and Sichuan had three (Fig. S2, Table S8).

**Fig 5:**
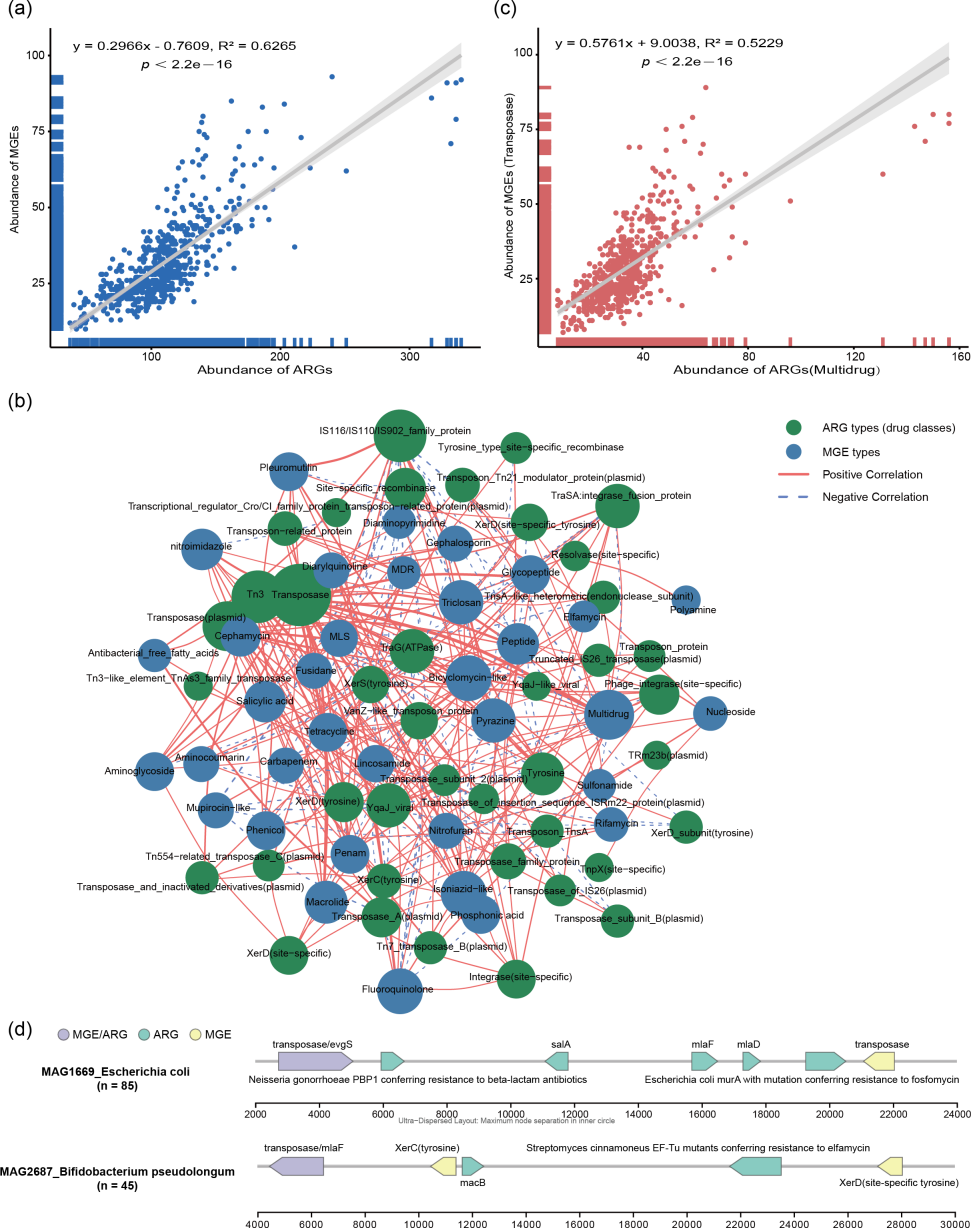
Correlation between ARGs and MGEs. (**a**) Scatter plot illustrating the linear correlation between total ARG and MGE abundance across 4,165 MAGs. (**b**) The network diagram based on Spearman correlation analysis showed a significant co-occurrence relationship between ARGs and MGEs (| r | > 0.1, *P* < 0.05). The green circle represents ARG types (drug classes), and the blue circle represents MGE types. The node size is proportional to the number of connections. The red solid line indicates a positive correlation, the blue-dotted line indicates a negative correlation, and the thick line is proportional to the absolute value strength of the correlation coefficient. (**c**) Correlation of multidrug ARG abundance and transposase MGE abundance. (**d**) ARGs and MGEs in *Escherichia coli* (MAG1669) and *Bifidobacterium pseudolongum* (MAG2687). The x-axis represents the position of genes within the continuous sequence. The direction of arrows indicates the strand on which the gene is located. Right arrows denote genes on the sense strand, while left arrows denote genes on the antisense strand. Numbers in parentheses on the left indicate the count of that gene in the MAG annotation.

To assess the relationship between ARGs and MGEs, we analyzed the correlation between their relative abundances across MAGs. A significant positive linear correlation was observed (R² = 0.6265, *P* < 2.2e–16), indicating that higher MGE count tended to be associated with a greater number of ARGs (Fig. 5a). A network diagram and heatmap showing correlations between specific ARG classes and MGE types further revealed strong associations (Fig. 5b; Fig. S3). Independent network analyses conducted in each province yielded consistent results (Fig. S4), highlighting the important role MGEs played in ARG dissemination. In particular, multidrug resistance genes showed a robust positive correlation with transposase genes (R² = 0.5229, *P* < 2.2e–16) (Fig. 5c).

To assess the risk of antibiotic resistance gene transmission at the strain level, we analyzed the distribution of MGEs within the genomes of *E. coli* (MAG1669) and the probiotic *B. pseudolongum* (MAG2687). Our findings revealed that transposases, as the most prevalent MGEs, were directly adjacent to various types of ARGs across multiple genomic regions. Notably, physical colocalization of ARGs and mobile genetic elements was prevalent across both genomes. A 2.2 kb region in MAG1669 harbored a high-density gene cluster containing six ARGs and two transposase genes, with one transposase gene colocalizing with the *evgS* gene (Fig. 5d). In a 2.6 kb region of MAG2687, a transposase gene was found colocalized with the *mlaF* gene, flanked by *XerC* and *XerD* site-specific recombinase genes (Fig. 5d). These findings suggest that in the aforementioned strains, MGEs may enhance the risk of horizontal transfer of ARGs.

## DISCUSSION

This study presents a comprehensive investigation of antibiotic resistance within the pig gut microbiome, utilizing metagenomic sequencing of 120 fecal samples collected from four Chinese provinces: Yunnan, Guizhou, Sichuan, and Jiangsu. Compared to the recently released integrated analysis workflow EasyMetagenome (49), this study employed a multi-tool integration approach and stringent quality control strategy, successfully assembling 4,165 medium-to-high-quality MAGs, of which 636 met high-quality standards (completeness ≥ 90%, contamination ≤ 5%). This data set expands the existing catalog of porcine gut MAGs (25, 50), providing robust genomic evidence for understanding the distribution and transmission characteristics of antimicrobial resistance in the pig gut within free-range environments.

The mammalian gut microbiota, comprising trillions of microorganisms, plays a pivotal role in host health by contributing to intestinal development, metabolism, and immunoregulation (51). The pig gut microbiome is dominated by specific bacterial phyla. In our study, Bacillota_A, Bacillota, Bacteroidota, Actinomycetota, and Spirochaetota were highly abundant. These phyla are known to perform essential physiological functions. For instance, Bacteroidota specializes in degrading complex carbohydrates, thereby producing short-chain fatty acids (SCFAs) that support gut integrity and energy metabolism (52, 53). Actinomycetota contributes to microbial stability and immune modulation through the production of bioactive compounds (54). The dominance of these phyla underscores their importance in maintaining gut health. Additionally, differences exist in the gut microbiota composition among pigs from different provinces. MAG1943 (*Lactobacillus amylovorus*) is enriched in Guizhou. This bacterium possesses strong amylolytic capabilities, secreting multiple enzymes to degrade starch and other polysaccharides while enhancing intestinal barrier function (55). This phenomenon may be linked to the local diet structure, which predominantly consists of starchy agricultural byproducts (56). Methanobrevibacter_A was most prevalent in Sichuan, Limosilactobacillus was elevated in Jiangsu, and Clostridium showed high detection rates in Yunnan. These regional variations may reflect the influence of geographical environments, farming practices, and locally distinctive agricultural methods on the shaping of pig gut microbial communities (57). Functional annotations via the KEGG and COG revealed that metabolic processes, especially those related to carbohydrate metabolism, were the most enriched pathways. This aligns with the dietary structure and gut environment of pigs (58). Furthermore, CAZy annotations highlighted the prevalence of GH and GT enzymes, which are essential for carbohydrate degradation and energy harvesting (59), reinforcing the metabolic specialization of the pig gut microbiota.

In 2017, the worldwide usage of veterinary antibiotics stood at approximately 93,309 tons, with projections indicating a rise to about 105,596 tons by 2030 (60, 61). Despite the ban on utilizing veterinary antibiotics for growth promotion purposes in Europe since 2005 and more recently in China, their application persists, particularly in developing nations (62). We identified 152 distinct ARG types spanning 35 drug resistance classes. In line with previous (63), multidrug, glycopeptide, peptide, and tetracycline resistance genes were the most prevalent. The pervasive occurrence of multidrug resistance genes may heighten the intricacy of managing intestinal infections and engender more extensive public health concerns (64). Notably, among the top 10 MAGs ranked by ARG counts, half were identified as *E. coli*. These *E. coli* exhibit high abundance and diversity in livestock and poultry manure and are widely recognized as significant reservoirs and vectors for ARGs (65). The high prevalence of ARGs in these strains underscores the challenges posed by their multidrug resistance in treating intestinal infections (66). Although people worldwide tend to consume probiotic products daily for their health benefits (67), this study also detected ARGs in probiotics such as *Bifidobacterium pseudolongum* and *Anaerobutyricum hallii*. This indicates that even beneficial microorganisms may serve as reservoirs for ARGs and potentially participate in the transmission of resistance genes (68). Differences exist in the ARG profiles of pig gut microbiota across different regions. Higher numbers of ARGs were observed in the gut microbiomes of pigs from Guizhou, Sichuan, and Jiangsu, which may be closely related to local agricultural practices, antibiotic usage patterns, and environmental conditions (69, 70). Jiangsu has a more developed economy and employs more intensive farming practices compared to other provinces (71). This intensive farming approach is often associated with higher antibiotic usage rates, potentially leading to elevated levels of ARGs in the pig gut microbiota (72). Guizhou possesses a high number of unique antibiotic resistance gene types. Guizhou’s terrain is characterized by undulating mountains and significant elevation differences and suffers from severe farmland fragmentation (73). This complex topography has shaped an agricultural production model dominated by small-scale family farms. Such small-scale farming often lacks effective disease monitoring and control systems, leading to the routine long-term use of antibiotics at low doses as a preventive measure (17, 74). This non-systematic antibiotic use may increase the prevalence of antibiotic-resistant genes within the gut microbiota of animals (75). In addition, Guizhou’s subtropical humid climate, with its high precipitation and humidity, fosters microbial proliferation, thereby driving the transmission and accumulation of ARGs through enhanced horizontal gene transfer (76). Sampling sites in Yunnan were characterized by a higher average altitude (2,232.00 m), extensive forest coverage, and richer vegetation types compared to other provinces (average altitude: 678.11 m) (77). These unique environmental conditions may be the reason for the small number of ARGs in Yunnan (78).

The connection between ARGs and MGEs is critical for assessing the potential for ARG dissemination within microbial ecosystems (79). Horizontal gene transfer, largely mediated by MGEs, is widely recognized as a primary pathway through which bacteria can acquire ARGs (80). We detected multiple types of MGEs in all fecal samples, of which transposases were the predominant type. Transposases are key enzymes that mediate the movement of transposons. MGEs are proposed to facilitate the transfer of ARGs across diverse bacterial taxa, thereby contributing to the persistence and spread of resistance traits in microbial communities (81). Li et al.’s study showed that the abundance of ARGs was positively correlated with that of transposase genes (82). This correlation provides indirect evidence, suggesting that transposable elements may mediate the spread of ARGs. Together, these findings lend further support to the crucial role HGT is likely to play in shaping drug resistance dynamics (83). Regional differences in MGE diversity may help explain the uneven distribution of ARGs among different regions and individuals (84). Furthermore, under antibiotic selection pressure, the presence of MGEs may drive the rapid evolution and adaptation of microorganisms, potentially exacerbating the challenge of antibiotic resistance further (85). Therefore, an intervention strategy targeting MGEs could be an effective method of controlling the spread of antibiotic resistance (86).

A study on health underscores that human health is inextricably linked to animal health and the ecological environment. Integrating multi-omics technologies can advance the concept of “One Health” for both humans and the environment (87). The metabolic rate of antibiotics in animals is low, and most of them (about 40%–90%) are excreted in the form of original drugs or metabolites through feces and urine (88). In China, pork is the main food source, accounting for more than 60% of daily meat intake, more than poultry consumption (89). Given that the free-range pig farms studied are located adjacent to human residential areas, we infer that ARGs may pose a potential risk of direct transmission to humans through the food chain or indirect transmission via fecal contamination of the environment (90, 91). However, our analysis is limited to fecal samples from these animals and does not include human or environmental samples. Therefore, it cannot provide direct evidence. Future research should incorporate these additional samples to gain a fuller understanding of antibiotic resistance genes and their transmission dynamics. Additionally, this study revealed the potential role of specific strains in spreading antibiotic resistance. However, the existing data are insufficient to confirm the origin and transfer mechanism of ARGs in these strains with certainty. Further research is needed to clarify these aspects and draw more definitive conclusions about the risks posed by these microorganisms.

### Conclusion

The large-scale assembly of MAGs from pig fecal samples has substantially advanced our understanding of the pig gut microbiome. Considering the widespread occurrence of antibiotic resistance, it is crucial to identify the origins and transmission pathways of ARGs. This study provides valuable genomic reference data for the porcine gut microbiota, revealing the complex dynamics between the host, microbial community, and environmental factors. Our findings underscore the importance of considering both microbial diversity and regional context when devising strategies to combat antibiotic resistance and enhance livestock health. Future research should focus on elucidating the specific roles of identified microbes and ARGs, as well as the molecular mechanisms regulating their interactions and transmission dynamics.

## Data Availability

The original data presented in this study are openly available in the NCBI (https://www.ncbi.nlm.nih.gov/bioproject/PRJNA1039141) under Bioproject accession number PRJNA1039141.

## SUPPLEMENTAL MATERIAL

Supplemental figures spectrum.02407-25-s0001.docx Supplemental figures.

Table S1 spectrum.02407-25-s0002.xlsx Metagenome-assembled genomes data summary of 120 samples.

Table S2 spectrum.02407-25-s0003.xlsx Binning and assembly results evaluation form.

Table S3 spectrum.02407-25-s0004.xlsx MAGs relative abundance.

Table S4 spectrum.02407-25-s0005.xlsx Summary of gene function annotations.

Table S5 spectrum.02407-25-s0006.xlsx Statistical table of drug resistance gene classification-1 and 2.

Table S6 spectrum.02407-25-s0007.xlsx ARGs statistics for MAG specific to each province.

Table S7 spectrum.02407-25-s0008.xlsx Summary of movable components.

Table S8 spectrum.02407-25-s0009.xlsx MGEs statistics for MAG specific to each province.

ASM does not own the copyrights to Supplemental Material that may be linked to, or accessed through, an article. The authors have granted ASM a non-exclusive, world-wide license to publish the Supplemental Material files. Please contact the corresponding author directly for reuse.
