# Supplementary material for: Insights into antibiotic resistomes from gut metagenome-assembled genomes of the free-range pigs
Source: Microbiol Spectr. 2026 Feb 27;14(4):e02407-25. doi: 10.1128/spectrum.02407-25 (PMC13055299; doi:10.1128/spectrum.02407-25)
Supplement: Supplemental figures [file spectrum.02407-25-s0001.docx]

**Supplementary Figures**


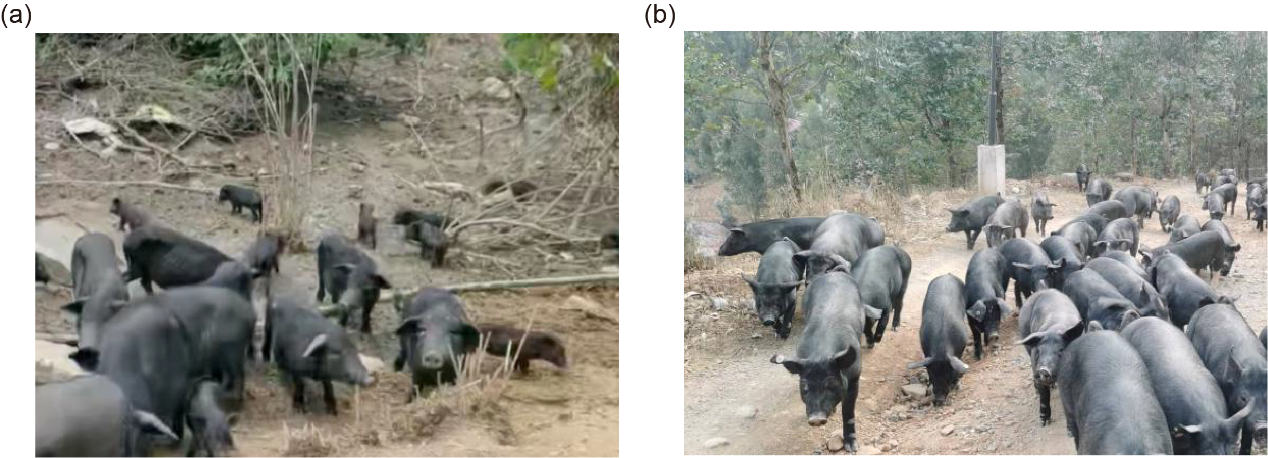


**Supplementary Fig. S1** An actual sampling environment. (a) An actual sampling environment from villages in Guizhou. (b) An actual sampling environment from villages in Sichuan. Fecal samples were taken from the pigs' farms that belong to the villagers. These farms are generally placed close to the villagers' houses.


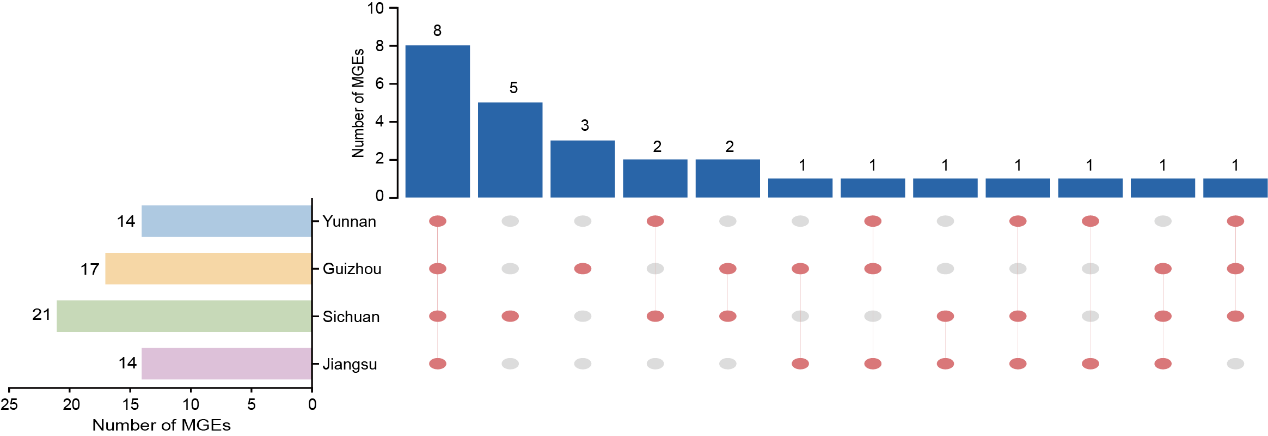


**Supplementary Fig. S2** Upset plot showing distribution and overlap of MGE subtypes among provinces. Vertical bars represent shared subtypes; dot matrix indicates combinations; horizontal bars show total MGE subtype counts per province.


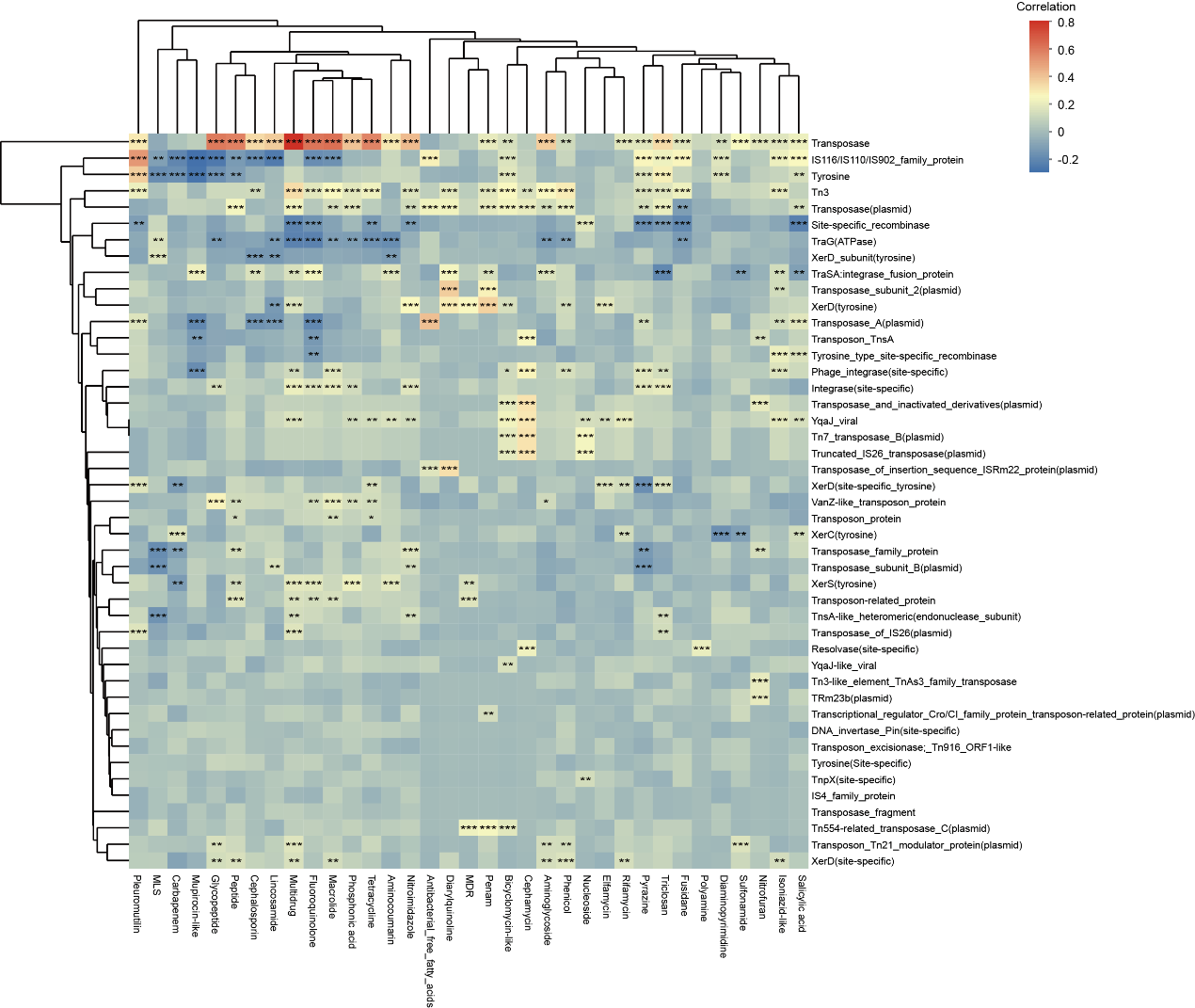


**Supplementary Fig. S3** Heatmap showing correlation coefficients between MGE types and ARG types (drug classes). Significance is indicated by: **p* < 0.05; ***p* < 0.01; ****p* < 0.001.


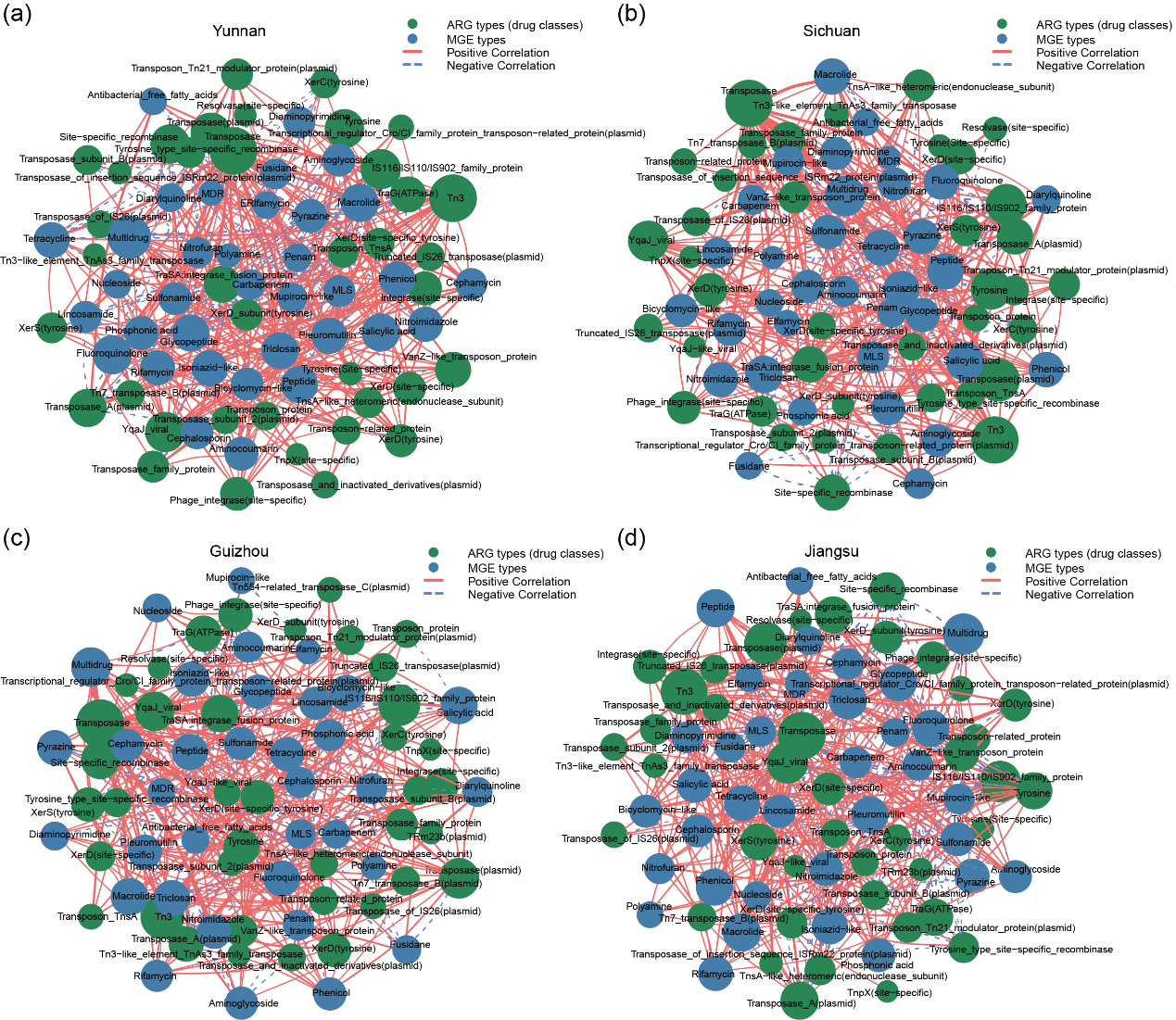


**Supplementary Fig. S4** Co-occurrence networks of ARGs and MGEs across four provinces. Networks were constructed based on Spearman’s correlation analysis (|r| > 0.1, *p* < 0.05). Green nodes represent ARG types (drug classes), and blue nodes represent MGE types. Node size is proportional to the number of connections. Red solid lines indicate positive correlations, blue dashed lines indicate negative correlations, and line thickness is proportional to the absolute value of the correlation coefficient. Panels show networks for (a) Yunnan Province, (b) Sichuan Province, (c) Guizhou Province, and (d) Jiangsu Province.
